# Torsion of an intrahydrocelic sac in a child: A case report

**DOI:** 10.1186/1757-1626-1-18

**Published:** 2008-06-16

**Authors:** Efstratios Christianakis, Nikolaos Paschalidis, Georgios Filippou, Maria Chorti, Nikolaos Andromanakos, Michael Pitiakoudis, Spiros Rizos, Dimitrios Filippou

**Affiliations:** 1 Department of Paediatric Surgery, Penteli's Children's Hospital, Athens, Greece; 2 First Department of General Surgery, Piraeus General Hospital "Tzaneio", Piraeus-Athens, Greece; 3 Department of Pathology, Athens General Hospital "Sismanoglion", Brilisia, Athens, Greece

## Abstract

We report the case of a 3-yr-old boy who presented an acute right hydrocele. A rapid scrotal swelling under tension developed the first hours and the child complained for discomfort especially during palpation of the scrotum. Three days later, surgical exploration revealed an incomplete torsion of a communicated and pedunculated peritoneal sac arising from the tunica vaginalis testis.

The present case report represents the eleventh report of torsion of processus vaginalis saccular protrusion in the literature, being unique due to painless hematocele.

## Background

In the patent processus vaginalis the containing fluid or any of the intraabdominal organs is characterized as hydrocele or hernia respectively. Hernia or hydroceles sacs as well as the rare saccular protrusion of processus vaginalis (SPPV) are peritoneal protrusions. Histologically a single layer of mesothelial origin cells, lining the inner cyst surface and loose connective tissue in the parietals may be present. In several cases the parietals of a congenital hernia sac may contain some muscle fibers. In the literature only fourteen similar complicated SPPV cases in children [1-10] and one more in an adult woman have been presented furthermore [10].

## Case presentation

Our case refers to a 3-year-old boy who presented to the emergency department, because of an acute sudden swelling of the right scrotum. Because of the swelling the child felt uncomfortable in palpation. The clinical examination revealed a trans-illuminated poorly encysted big hydrocele like a common cyst of spermatic cord in tension. The cyst was afebrile having bilateral cremasteric reflexes. As an infant, he had a big scrotal hydrocele on the right side, which gradually disappeared until the 15th month of his life. Blood and urine laboratory tests, in exception of elevated white blood cells (WBC: 15,000/μl) were normal.

**Figure 1:**
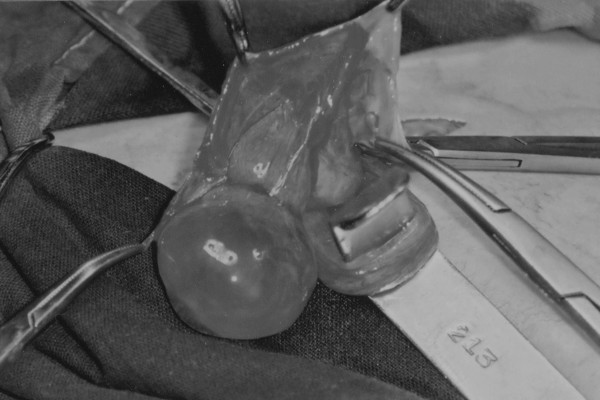
Twisted but not necrotized SPPV, with a blood-colored external wall. Mosquito forceps hang on the wall of the main hydrocele sac.

**Figure 2:**
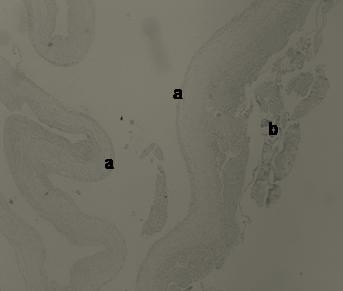
H-E × 40. Histological view of the intrahydrocelic sac revealing the internal surface lining of the mesothelial cells (a) and the attached fibrin secondary to the torsion (b).

The preoperative clinical and ultrasound diagnosis suggested hydrocele in tension and the child was operated three days later. Surgical exploration revealed a hematocele, with bloody fluid accumulation mainly in the tunica vaginalis testis. The SPPV was hanging from a twisted pedicle, arising from the internal parietal layer of tunica vaginalis. Dissection and resection of the hydrocele sac, including the SPPV was performed. The appearance of the twisted SPPV was non-translucent (figures 1), instead of another normal no-twisted SPPV. The histological examination of the twisted SPPV revealed a lining of mesothelial cells and the presence of fibrin, secondary to the torsion (figure 2). The patient presented no postoperative complications and discharged the next day.

## Discussion

The SPPVs, which are usually referred as intrahernial hydroceles or intrahydrocelic hernias, are fluid-filled sacs lying entirely within the hernias. Intrahernial or intrahydrocelic sacs are a more accurate term, because these entities containing fluid contents and are free hanging. They seem like simple serous cysts, with a short or long pedunculated stalk, communicating through a small hole, as in our case in the hanging part with the intrahydrocelic space.

The first who described a pedunculated cyst within the hernia sac, a**s **a partial distal intussusception of a patent processus vaginalis, in a child, was Dr. Lockwood in 1896 [1]. Lockwood had initially the same wrong impression to the authors of the present case report. Bloom DA et al and Perez LM et al on 1993 and 1999 respectively described three cases of intussuscepted patent processus vaginalis which was twisted [1,2].

In the literature only eight cases referring to "torsion of an inguinal hernia" [2-7] and three more ''to torsion of a communicated hydrocele or benign cyst'' have been presented [8-10]. The vast majority of the presented cases refer to children except a unique report from Russia which describes torsion of an intrahernial cyst in an adult female patient [10]. The containing fluid in all these congenital origin sacs was serous or bloody.

The SPPV most frequently lie just proximal to the scrotal inlet or close to the testis and it can produce discomfort or acute scrotum in cases that is tearing. For this reason they must be included in the differential diagnosis of suddenly appearing persistent hydroceles or acute scrotum in children. The severity of symptoms depends on the cyst's size and the existence and the pattern of the concomitant torsion. Long pedunculated small cysts are more predisposing in complete torsion. This fact is facilitating from the gravity, since the cyst is hanging from a firm point towards the bottom of the hydrocele sac, exactly as the clapper bell. Eleven out of the thirteen reported cases were presented with painful scrotal swellings, but two were painless enlargements, as in our case [7-9]. This is may be a possible explanation for the great number of undiagnosed transiently twisted SPPVs. In addition, these must be differentiated from the inguinal cystic lymphangiomas.

The preoperative clinical and ultrasound diagnosis in our case was difficult. The cyst was occupying almost the entire anatomical space of the tunica vaginalis without acute scrotum, because of partially twisting. In such cases bleeding may occur mainly from the external parietal vessels lying next to the cyst, although it is difficult to cause necrosis of the cyst. In all of the reported cases the patients did not present nausea, vomiting or abdominal pain, symptoms that are very common in children with testicular torsion.

## Conclusion

In conclusion, the SPPVs are rare inguinal-scrotal pedunculated sacs which containing serous fluid. These are rather types of communicated, coexisting and non-classified hydroceles instead of hernias that lie in a hydrocelic or hernia sac. In very rare cases SPPVs can twist producing either uncomforting like hydroceles in tension or symptoms of acute scrotum.

## Consent

Written consent was obtained from the patient, a copy is available from the editor in chief.

## Authors' contributions

CE, FD, FG, CM, AN, PM, RS, and PN contributed equally to the patient's therapy, writing the present case report and approving it.
